# Supplementary material for: TROAP Promotes the Proliferation, Migration, and Metastasis of Kidney Renal Clear Cell Carcinoma with the Help of STAT3
Source: Int J Mol Sci. 2023 Jun 2;24(11):9658. doi: 10.3390/ijms24119658 (PMC10253451; doi:10.3390/ijms24119658)
Supplement: Supplementary file 1 [file ijms-24-09658-s001.zip › Supplementary Figure.pdf]

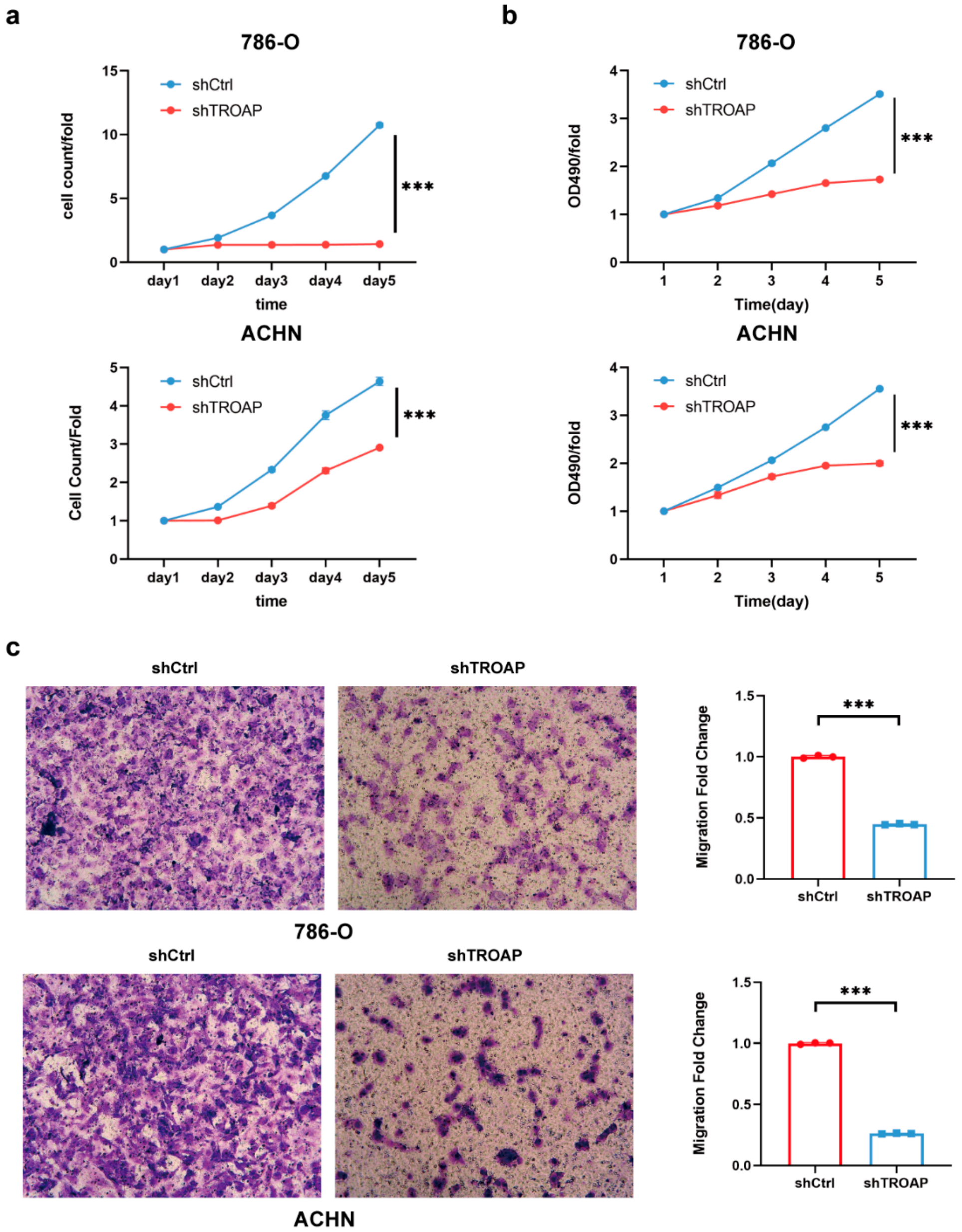

Figure S1. TROAP function in 786-O and ACHN cell line. (a, b) Cell proliferation in the shCtrl and shTROAP groups was determined using Celigo and MTT assays. (c) 786-O and ACHN cell migration due toTROAP inhibition was investigated by Transwell assay. The original magnification is 100× ***$P < 0.001$.

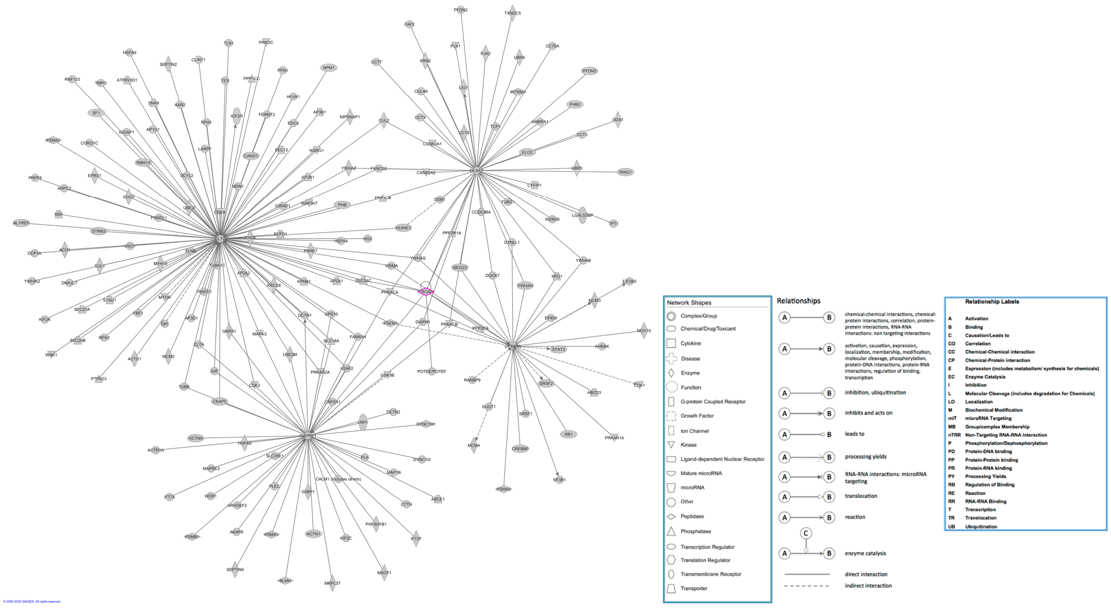

Figure S2. The gene interaction network was based on our results.
