# Supplementary material for: TROAP Promotes the Proliferation, Migration, and Metastasis of Kidney Renal Clear Cell Carcinoma with the Help of STAT3
Source: Int J Mol Sci. 2023 Jun 2;24(11):9658. doi: 10.3390/ijms24119658 (PMC10253451; doi:10.3390/ijms24119658)
Supplement: Supplementary file 1 [file ijms-24-09658-s001.zip › Supplementary Table.pdf]

Supplementary Table S1 Raw data files for 72 paired samples.

No	bcr_patient_barcode	year_of_initial_pathologic_diagnosis	pathologic_T	pathologic_N	pathologic_M	pathologic_stage	vital_status	days_to_death	age_at_initial_pathologic_diagnosis
1	TCGA-A3-3358	2005	T1a	N0	M0	Stage I	Alive	NA	57
2	TCGA-A3-3387	2006	T1a	N0	M0	Stage I	Alive	NA	49
3	TCGA-B0-4700	2004	T4	NX	M1	Stage IV	Dead	1980	60
4	TCGA-B0-4712	2006	T3a	NX	M1	Stage IV	Dead	1337	76
5	TCGA-B0-5402	2010	T4	NX	M0	Stage IV	Alive	NA	64
6	TCGA-B0-5690	2004	T1b	NX	M0	Stage I	Alive	NA	53
7	TCGA-B0-5691	2002	T1a	N0	M0	Stage I	Alive	NA	66
8	TCGA-B0-5694	2008	T3a	N0	M0	Stage III	Dead	480	71
9	TCGA-B0-5696	2007	T3a	N0	M0	Stage III	Alive	NA	69
10	TCGA-B0-5697	2007	T1a	N0	M0	Stage I	Alive	NA	50
11	TCGA-B0-5699	2003	T1	N0	M0	Stage I	Alive	NA	53
12	TCGA-B0-5701	2007	T3b	N0	M0	Stage III	Alive	NA	65
13	TCGA-B0-5703	2009	T1b	N0	M0	Stage I	Alive	NA	73
14	TCGA-B0-5705	2002	T1	N0	M0	Stage I	Alive	NA	65
15	TCGA-B0-5706	2004	T2	N0	M0	Stage II	Alive	NA	45
16	TCGA-B0-5709	2003	T3a	NX	M0	Stage III	Alive	NA	62
17	TCGA-B0-5711	2004	T3b	NX	M0	Stage III	Alive	NA	50
18	TCGA-B0-5712	2004	T2	N0	M1	Stage IV	Alive	NA	68
19	TCGA-B2-5636	2010	T1a	NX	M0	Stage I	Alive	NA	79
20	TCGA-B2-5641	2010	T1a	N0	M0	Stage I	Alive	NA	79
21	TCGA-B8-4619	2010	T1a	N0	M0	Stage I	Alive	NA	58
22	TCGA-B8-4620	2010	T3a	N0	M0	Stage III	Alive	NA	70
23	TCGA-B8-4622	2010	T3a	N0	M1	Stage IV	Alive	NA	57
24	TCGA-B8-5549	2010	T1b	N0	M0	Stage I	Alive	NA	53
25	TCGA-B8-5552	2010	T1b	NX	M0	Stage I	Alive	NA	41
26	TCGA-CJ-5672	2004	T1a	NX	M0	Stage I	Dead	2190	84
27	TCGA-CJ-5676	2004	T3b	NX	M0	Stage III	Alive	NA	47
28	TCGA-CJ-5677	2004	T3a	NX	M1	Stage IV	Dead	782	54
29	TCGA-CJ-5678	2003	T2b	N0	M1	Stage IV	Dead	574	62
30	TCGA-CJ-5679	2003	T3b	NX	M0	Stage III	Dead	679	73
31	TCGA-CJ-5680	2003	T3a	NX	M1	Stage IV	Dead	768	65
32	TCGA-CJ-5681	2003	T3a	NX	M1	Stage IV	Dead	552	44
33	TCGA-CJ-5689	2005	T1b	NX	M0	Stage I	Dead	1620	90
34	TCGA-CJ-6030	2003	T1a	N0	M0	Stage I	Dead	2299	65
35	TCGA-CJ-6033	2004	T3a	N0	M1	Stage IV	Dead	224	54
36	TCGA-CW-5580	2003	T3a	NX	M1	Stage IV	Dead	1964	73
37	TCGA-CW-5581	2003	T1b	NX	M0	Stage I	Alive	NA	44
38	TCGA-CW-5584	2003	T3b	N1	M0	Stage III	Dead	164	74

39	TCGA-CW-5585	2003	T3b	N0	M1	Stage IV	Alive	NA	51
40	TCGA-CW-5587	2003	T3b	N0	M0	Stage III	Alive	NA	62
41	TCGA-CW-5589	2004	T1a	NX	M0	Stage I	Alive	NA	52
42	TCGA-CW-5591	2004	T3a	N0	M1	Stage IV	Alive	NA	56
43	TCGA-CW-6087	2003	T3a	N1	M1	Stage IV	Dead	41	61
44	TCGA-CW-6088	2003	T1b	N0	M0	Stage I	Alive	NA	60
45	TCGA-CW-6090	2003	T1b	NX	M0	Stage I	Alive	NA	68
46	TCGA-CZ-4863	2006	T3b	N0	M0	Stage III	Alive	NA	51
47	TCGA-CZ-4864	2002	T2	N0	M0	Stage II	Dead	2830	86
48	TCGA-CZ-4865	2006	T1a	NX	M0	Stage I	Dead	166	70
49	TCGA-CZ-5451	2006	T2	N0	M0	Stage II	Alive	NA	74
50	TCGA-CZ-5452	2006	T2	N0	M0	Stage II	Alive	NA	69
51	TCGA-CZ-5453	2006	T2	NX	M0	Stage II	Alive	NA	67
52	TCGA-CZ-5454	2006	T2	N0	M1	Stage IV	Dead	722	63
53	TCGA-CZ-5455	2006	T3b	NX	M1	Stage IV	Dead	561	63
54	TCGA-CZ-5456	2006	T2	N0	M0	Stage II	Alive	NA	57
55	TCGA-CZ-5457	2006	T3a	NX	M0	Stage III	Alive	NA	62
56	TCGA-CZ-5458	2006	T3a	NX	M0	Stage III	Alive	NA	43
57	TCGA-CZ-5461	2006	T1b	NX	M1	Stage IV	Dead	330	52
58	TCGA-CZ-5462	2007	T1b	NX	M1	Stage IV	Dead	311	83
59	TCGA-CZ-5463	2007	T2	NX	M0	Stage II	Alive	NA	76
60	TCGA-CZ-5465	2007	T3b	NX	M0	Stage III	Alive	NA	76
61	TCGA-CZ-5466	2007	T3a	NX	M0	Stage III	Alive	NA	67
62	TCGA-CZ-5467	2007	T3a	N0	M0	Stage III	Dead	73	86
63	TCGA-CZ-5468	2007	T3b	NX	M1	Stage IV	Dead	59	84
64	TCGA-CZ-5469	2007	T2	N0	M0	Stage II	Dead	946	41
65	TCGA-CZ-5470	2008	T2	N0	M0	Stage II	Alive	NA	72
66	TCGA-CZ-5982	2005	T1a	NX	M0	Stage I	Alive	NA	59
67	TCGA-CZ-5984	2005	T1b	N0	M0	Stage I	Alive	NA	51
68	TCGA-CZ-5985	2006	T2	N0	M0	Stage II	Alive	NA	58
69	TCGA-CZ-5986	2006	T1	N0	M0	Stage I	Alive	NA	61
70	TCGA-CZ-5987	2006	T3b	NX	M1	Stage IV	Dead	445	60
71	TCGA-CZ-5988	2006	T1b	N0	M0	Stage I	Alive	NA	38
72	TCGA-CZ-5989	2006	T2	N0	M0	Stage II	Alive	NA	60

Supplementary Table S2 Sequences of primers used for qPCR.

**mRNA**	**Primer**	
	**FORWARD**	**REVERSE**
**GAPDH**	TGACTTCAACAGCGACACCCA	CACCCTGTTGCTGTAGCCAAA
**TROAP**	CCTCCGGGGTGTATCTCCTAC	ACGGCGCACGATGTAACAG
**STAT3**	AGAAGGACATCAGCGGTAAG	CCTTGGGAATGTCAGGATAGAG

Supplementary Table S3 Lentiviral sequence information.

Lentiviral	Sequence
**LV-TROAP**	CCAACCGGTCGCCACCATGGACTACAAAGACCATGACGGTGATTAT<br>AAAGATCATGACATCGATTACAAGGATGACGATGACAAGTCAGCTA<br>GCGTTATTGAATTCATGACCACCCGGCAAGCCACGAAGGATCCCCT<br>CCTCCGGGGTGTATCTCCTACCCCTAGCAAGATTCCGGTACGCTCTC<br>AGAAACGCACGCCTTTCCCCACTGTTACATCGTGCGCCGTGGACCA<br>GGAGAACCAAGATCCAAGGAGATGGGTGCAGAAACCACCGCTCAA<br>TATTCAACGCCCCCTCGTTGATTCAGCAGGCCCCAGGCCGAAAGCC<br>AGGCACCAGGCAGAGACATCACAAAGATTGGTGGGGATCAGTCAG<br>CCTCGGAACCCCTTGGAAGAGCTCAGGCCTAGCCCTAGGGGTCAAA<br>ATGTGGGGCCTGGGCCCCCTGCCCAGACAGAGGCTCCAGGGACCAT<br>AGAGTTTGTGGCTGACCCTGCAGCCCTGGCCACCATCCTGTCAGGTG<br>AGGGTGTGAAGAGCTGTCACCTGGGGCGCCAGCCTAGTCTGGCTAA<br>AAGAGTACTGGTTCGAGGAAGTCAGGGAGGCACCACCCAGAGGGT<br>CCAGGGTGTTCGGGCCTCTGCATATTTGGCCCCCAGAACCCCCACCC<br>ACCGACTGGACCCTGCCAGGGCTTCCTGCTTCTCTAGGCTGGAGGG<br>ACCAGGACCTCGAGGCCGGACATTGTGCCCCCAGAGGCTACAGGCT<br>CTGATTTCACCTTCAGGACCTTCCTTTCACCCTTCCACTCGCCCCAGT<br>TTCCAGGAGCTAAGAAGGGAGACAGCTGGCAGCAGCCGGACTTCA<br>GTGAGCCAGGCCTCAGGATTGCTCCTGGAGACCCCAGTCCAGCCTG<br>CTTTCTCTCTTCCTAAAGGAGAACGCGAGGTTGTCACTCACTCAGAT<br>GAAGGAGGTGTGGCCTCTCTTGGTCTGGCCCAGCGAGTACCATTAA<br>GAGAAAACCGAGAAATGTCACATACCAGGGACAGCCATGACTCCC<br>ACCTGATGCCCTCCCCTGCCCCTGTGGCCCAGCCCTTGCCTGGCCAT<br>GTGGTGCCATGTCCATCACCCTTTGGACGGGCTCAGCGTGTACCCTC<br>CCCAGGCCCTCCAACTCTGACCTCATATTCAGTGTTGCGGCGTCTCA<br>CCGTTCAACCTAAAACCCGGTTCACACCCATGCCATCAACCCCCAG<br>AGTTCAGCAGGCCCAGTGGCTGCGTGGTGTCTCCCCTCAGTCCTGCT<br>CTGAAGATCCTGCCCTGCCCTGGGAGCAGGTTGCCGTCCGGTTGTTT<br>GACCAGGAGAGTTGTATAAGGTCACTGGAGGGTTCTGGGAAACCAC<br>CGGTGGCCACTCCTTCTGGACCCCACTCTAACAGAACCCCCAGCCTC<br>CAGGAGGTGAAGATTCAACGCATCGGTATCCTGCAACAGCTGTTGA<br>GACAGGAAGTAGAGGGGCTGGTAGGGGGCCAGTGTGTCCCTCTTAA<br>TGGAGGCTCTTCTCTGGATATGGTTGAACTTCAGCCCCTGCTGACTG<br>AGATTTCTAGAACTCTGAATGCCACAGAGCATAACTCTGGGACTTC<br>CCACCTTCCTGGACTGTTAAAACACTCAGGGCTGCCAAAGCCCTGTC<br>TTCCAGAGGAGTGTGGGGAACCACAGCCCTGCCCTCCGGCAGAGCC<br>TGGGCCCCCAGAGGCCTTCTGTAGGAGTGAGCCTGAGATACCAGAG<br>CCCTCCCTCCAGGAACAGCTTGAAGTACCAGAGCCCTACCCTCCAG<br>CAGAACCCAGGCCCCTAGAGTCCTGCTGTAGGAGTGAGCCTGAGAT<br>ACCGGAGTCCTCTCGCCAGGAACAGCTTGAGGTACCTGAGCCCTGC<br>CCTCCAGCAGAACCCAGGCCCCTAGAGTCCTACTGTAGGATTGAGC<br>CTGAGATACCGGAGTCCTCTCGCCAGGAACAGCTTGAGGTACCTGA

	GCCCTGCCCTCCAGCAGAACCCGGGCCCCTTCAGCCCAGCACCCAG
	GGGCAGTCTGGACCCCCAGGGCCCTGCCCTAGGGTAGAGCTGGGGG
	CATCAGAGCCCTGCACCCTGGAACATAGAAGTCTAGAGTCCAGTCT
	ACCACCCTGCTGCAGTCAGTGGGCTCCAGCAACCACCAGCCTGATC
	TTCTCTTCCCAACACCCGCTTTGTGCCAGCCCCCCTATCTGCTCACTC
	CAGTCTTTGAGACCCCCAGCAGGCCAGGCAGGCCTCAGCAATCTGG
	CCCCTCGAACCCTAGCCCTGAGGGAGCGCCTCAAATCGTGTTTAAC
	CGCCATCCACTGCTTCCACGAGGCTCGTCTGGACGATGAGTGTGCCT
	TTTACACCAGCCGAGCCCCTCCCTCAGGCCCCACCCGGGTCTGCACC
	AACCCTGTGGCTACATTACTCGAATGGCAGGATGCCCTGTGTTTCAT
	TCCAGTTGGTTCTGCTGCCCCCCAGGGCTCTCCATGAGAATTCTAGT
	TATTAATAGTAATCAATTACGGGGTCATTAGTTCATAGCCCATATAT
	GGAGTTCCGCGTTACATAACTTACGGTAAATGGCCCGCCTGGCT
**LV-STAT3**	GCCGTTTTTGGCTTTTTTGTTAGACGAAGCTTGGGCTGCAGGTCGAC
	TCTAGAGGATCCCGCCACCATGGCCCAATGGAATCAGCTACAGCAG
	CTTGACACACGGTACCTGGAGCAGCTCCATCAGCTCTACAGTGACA
	GCTTCCCAATGGAGCTGCGGCAGTTTCTGGCCCCTTGGATTGAGAGT
	CAAGATTGGGCATATGCGGCCAGCAAAGAATCACATGCCACTTTGG
	TGTTTCATAATCTCCTGGGAGAGATTGACCAGCAGTATAGCCGCTTC
	CTGCAAGAGTCGAATGTTCTCTATCAGCACAATCTACGAAGAATCA
	AGCAGTTTCTTCAGAGCAGGTATCTTGAGAAGCCAATGGAGATTGC
	CCGGATTGTGGCCCGGTGCCTGTGGGAAGAATCACGCCTTCTACAG
	ACTGCAGCCACTGCGGCCCAGCAAGGGGGCCAGGCCAACCACCCCA
	CAGCAGCCGTGGTGACGGAGAAGCAGCAGATGCTGGAGCAGCACC
	TTCAGGATGTCCGGAAGAGAGTGCAGGATCTAGAACAGAAAATGA
	AAGTGGTAGAGAATCTCCAGGATGACTTTGATTTCAACTATAAAAC
	CCTCAAGAGTCAAGGAGACATGCAAGATCTGAATGGAAACAACCA
	GTCAGTGACCAGGCAGAAGATGCAGCAGCTGGAACAGATGCTCACT
	GCGCTGGACCAGATGCGGAGAAGCATCGTGAGTGAGCTGGCGGGG
	CTTTTGTCAGCGATGGAGTACGTGCAGAAAACTCTCACGGACGAGG
	AGCTGGCTGACTGGAAGAGGCGGCAACAGATTGCCTGCATTGGAGG
	CCCGCCCAACATCTGCCTAGATCGGCTAGAAAACTGGATAACGTCA
	TTAGCAGAATCTCAACTTCAGACCCGTCAACAAATTAAGAAACTGG
	AGGAGTTGCAGCAAAAAGTTTCCTACAAAGGGGACCCCATTGTACA
	GCACCGGCCGATGCTGGAGGAGAGAATCGTGGAGCTGTTTAGAAAC
	TTAATGAAAAGTGCCTTTGTGGTGGAGCGGCAGCCCTGCATGCCCA
	TGCATCCTGACCGGCCCCTCGTCATCAAGACCGGCGTCCAGTTCACT
	ACTAAAGTCAGGTTGCTGGTCAAATTCCCTGAGTTGAATTATCAGCT
	TAAAATTAAAGTGTGCATTGACAAAGACTCTGGGGACGTTGCAGCT
	CTCAGAGGATCCCGGAAATTTAACATTCTGGGCACAAACACAAAAG
	TGATGAACATGGAAGAATCCAACAACGGCAGCCTCTCTGCAGAATT
	CAAACACTTGACCCTGAGGGAGCAGAGATGTGGGAATGGGGGCCG
	AGCCAATTGTGATGCTTCCCTGATTGTGACTGAGGAGCTGCACCTGA
	TCACCTTTGAGACCGAGGTGTATCACCAAGGCCTCAAGATTGACCT

AGAGACCCACTCCTTGCCAGTTGTGGTGATCTCCAACATCTGTCAGA
TGCCAAATGCCTGGGCGTCCATCCTGTGGTACAACATGCTGACCAA
CAATCCCAAGAATGTAAACTTTTTACCAAGCCCCCAATTGGAACCT
GGGATCAAGTGGCCGAGGTCCTGAGCTGGCAGTTCTCCTCCACCAC
CAAGCGAGGACTGAGCATCGAGCAGCTGACTACACTGGCAGAGAA
ACTCTTGGGACCTGGTGTGAATTATTCAGGGTGTCAGATCACATGG
GCTAAATTTTGCAAAGAAAACATGGCTGGCAAGGGCTTCTCCTTCT
GGGTCTGGCTGGACAATATCATTGACCTTGTGAAAAAGTACATCCT
GGCCCTTTGGAACGAAGGGTACATCATGGGCTTTATCAGTAAGGAG
CGGGAGCGGGCCATCTTGAGCACTAAGCCTCCAGGCACCTTCCTGC
TAAGATTCAGTGAAAGCAGCAAAGAAGGAGGCGTCACTTTCACTTG
GGTGGAGAAGGACATCAGCGGTAAGACCCAGATCCAGTCCGTGGA
ACCATACACAAAGCAGCAGCTGAACAACATGTCATTTGCTGAAATC
ATCATGGGCTATAAGATCATGGATGCTACCAATATCCTGGTGTCTCC
ACTGGTCTATCTCTATCCTGACATTCCCAAGGAGGAGGCATTCGGA
AAGTATTGTCGGCCAGAGAGCCAGGAGCATCCTGAAGCTGACCCAG
GTAGCGCTGCCCCATACCTGAAGACCAAGTTTATCTGTGTGACACC
AACGACCTGCAGCAATACCATTGACCTGCCGATGTCCCCCCGCACTT
TAGATTCATTGATGCAGTTTGGAAATAATGGTGAAGGTGCTGAACC
CTCAGCAGGAGGGCAGTTTGAGTCCCTCACCTTTGACATGGAGTTG
ACCTCGGAGTGCGCTACCTCCCCCATGTGAGCTAGCCTGTGGAATGT
GTGTCAGTTAGGGTGTGGAAAGTCCCCAGGCTCCCCAGCAGGCAGA
AGTATGCAAAGCATGCATCTCAATTAGTCAGCAACCA

Yellow marker: Synonymous mutation of TGC to TGT

Supplementary Table S4 The protein identification results.

Database	Samples Name	Total spectra	PSM	Peptides	Protein groups
HomoSapiens	NC	9948	205	195	122
HomoSapiens	OE	19069	7331	6327	1526
